# A Randomized Controlled ERP Study on the Effects of Multi-Domain Cognitive Training and Task Difficulty on Task Switching Performance in Older Adults

**DOI:** 10.3389/fnhum.2017.00184

**Published:** 2017-04-12

**Authors:** Kristina Küper, Patrick D. Gajewski, Claudia Frieg, Michael Falkenstein

**Affiliations:** Aging Research Group, Leibniz Research Centre for Working Environment and Human FactorsDortmund, Germany

**Keywords:** cognitive training, task difficulty, aging, Stroop switch task, switch positivity, P3, P2, N2

## Abstract

Executive functions are subject to a marked age-related decline, but have been shown to benefit from cognitive training interventions. As of yet, it is, however, still relatively unclear which neural mechanism can mediate training-related performance gains. In the present electrophysiological study, we examined the effects of multi-domain cognitive training on performance in an untrained cue-based task switch paradigm featuring Stroop color words: participants either had to indicate the word meaning of Stroop stimuli (word task) or perform the more difficult task of color naming (color task). One-hundred and three older adults (>65 years old) were randomly assigned to a training group receiving a 4-month multi-domain cognitive training, a passive no-contact control group or an active (social) control group receiving a 4-month relaxation training. For all groups, we recorded performance and EEG measures before and after the intervention. For the cognitive training group, but not for the two control groups, we observed an increase in response accuracy at posttest, irrespective of task and trial type. No training-related effects on reaction times were found. Cognitive training was also associated with an overall increase in N2 amplitude and a decrease of P2 latency on single trials. Training-related performance gains were thus likely mediated by an enhancement of response selection and improved access to relevant stimulus-response mappings. Additionally, cognitive training was associated with an amplitude decrease in the time window of the target-locked P3 at fronto-central electrodes. An increase in the switch positivity during advance task preparation emerged after both cognitive and relaxation training. Training-related behavioral and event-related potential (ERP) effects were not modulated by task difficulty. The data suggest that cognitive training increased slow negative potentials during target processing which enhanced the N2 and reduced a subsequent P3-like component on both switch and non-switch trials and irrespective of task difficulty. Our findings further corroborate the effectiveness of multi-domain cognitive training in older adults and indicate that ERPs can be instrumental in uncovering the neural processes underlying training-related performance gains.

## Introduction

All planned goal-directed behavior is mediated by executive control functions, such as selective attention, working memory, the inhibition of irrelevant information or the selection and coordination of relevant task sets. Previous research has indicated that these functions are subject to a marked age-related decline beginning as early as in midlife (Li et al., 2004; Sander et al., 2012). Given the crucial role of executive control functions for activities of daily living, age-related deficits in this domain can be particularly detrimental to the well-being and autonomy of older adults (Burgess et al., 1998; Jonides et al., 2008). Cognitive functions show a remarkable degree of plasticity across the lifespan, however, and can hence benefit from different types of training interventions up to a very old age (Hertzog et al., 2008; Karbach and Schubert, 2013; for reviews see Kueider et al., 2012; Ballesteros et al., 2015).

Cognitive training regimen which focus on a single domain or task, such as working memory or task switching, have been shown to consistently improve performance in the trained task (for meta-analyses, see Karbach and Verhaeghen, 2014; Lampit et al., 2014b; Au et al., 2015). Transfer of such training gains to untrained tasks or everyday functioning appears to be more limited, however, and has been reported only in some cases (Karbach and Verhaeghen, 2014; Au et al., 2015), but not in others (Ball et al., 2002; Melby-Lervåg and Hulme, 2013; Melby-Lervåg et al., 2016).

It has been hypothesized that a substantial overlap between the processes underlying performance in the training task and those underlying performance in the transfer task is necessary for successful transfer to occur (Jonides, 2004; Dahlin et al., 2008; Lustig et al., 2009; Buschkuehl et al., 2012). In light of this, cognitive training interventions which focus not only on a single function but on multiple cognitive functions have recently been discussed as a more effective training measure which may potentially yield broader transfer effects (Gates and Valenzuela, 2010; Karbach, 2014). In keeping with this, cognitive training programs integrating multiple tasks have shown both near and far transfer effects to measures of perceptual processing, working memory updating, memory accuracy and reasoning (Mahncke et al., 2006; Wild-Wall et al., 2012; Walton et al., 2014; Baniqued et al., 2015). Moreover, Lampit et al. (2014a) reported transfer gains from a multi-domain training aimed at reasoning, memory, attention and visuo-spatial abilities to a bookkeeping task closely mirroring a real-world work scenario. In direct comparisons to single-domain interventions, multi-domain cognitive training has additionally been associated with more pronounced benefits in far transfer tasks measuring executive attentional control (Binder et al., 2016) and increased longevity of training-related performance benefits (Cheng et al., 2012).

Recent resting-state fMRI studies have been able to offer some insights into the neural processes which may mediate performance gains associated with multi-domain cognitive training: older adults who have undergone multi-domain cognitive training show increased neural lateralization and functional connectivity (Cao et al., 2016; Li et al., 2016; Luo et al., 2016; see also Binder et al., 2017, for similar electrophysiological data). Such brain activation patterns are commonly found in much younger adults suggesting that multi-domain cognitive training may be able to compensate age-related changes to neural connectivity at least to some degree. In keeping with this, multi-domain cognitive training has also been associated with a reduction in age-related cortical thinning in fronto-temporal areas (Kim et al., 2015; Jiang et al., 2016).

A drawback of these imaging studies is that they employed predominantly passive control groups. It is thus possible that the structural differences observed between training and control groups reflect differences in general activity rather than training-specific benefits (see Redick et al., 2013). Moreover, it is still relatively unclear what functional consequences the observed structural changes may have, especially for the crucial domain of executive control. In two previous studies, we thus compared middle-aged and older adults who had undergone multi-domain cognitive training to both active and passive control groups and examined event-related potential (ERP) indices of performance in a task switching paradigm (Gajewski and Falkenstein, 2012; Gajewski et al., 2017).

Task switching paradigms have the advantage of yielding indices of multiple distinct subcomponents of executive control. The paradigm requires participants to attend to two or more different tasks in distinct experimental blocks. In single blocks, participants always have to perform only one of the tasks whereas they have to flexibly switch between different tasks on a trial-by-trial basis in the mixed block. Mixed blocks thus feature stay trials on which the same task as in the preceding trial has to be performed and switch trials on which a different task has to be performed. In memory-based switch tasks, participants have to memorize a fixed task order for these mixed blocks. In cue-based paradigms, the task order is instead random and a cue preceding each target stimulus indicates which task is to be performed.

Despite the fact that both single and stay trials constitute task repetitions, performance on trials in the single block is usually better than on stay trials in the mixed block. These general switch costs or mixing costs have been interpreted as indexing the ability to maintain a task set in the context of a different, interfering task set. The ability to flexibly switch between tasks on a trial-by trial basis is instead reflected in specific or local switch costs, i.e., performance decrements in switch trials relative to stay trials (Allport et al., 1994; Rogers and Monsell, 1995; Meiran, 1996; see Kiesel et al., 2010, for a review). Age-comparative research has indicated that older adults show increased general switch costs relative to younger adults, but similar specific switch costs (Kramer et al., 1999; Kray and Lindenberger, 2000; Mayr, 2001). It thus appears that aging negatively affects the ability to simultaneously maintain and coordinate distinct task sets, but leaves task switching abilities relatively intact.

Our previous training studies have indicated that multi-domain cognitive training has the potential to compensate at least some of this age-related deficit and can lead to a reduction in general switch costs (Gajewski and Falkenstein, 2012; Gajewski et al., 2017). In our studies, these performance gains were accompanied by amplitude increases in three ERPs, the N2, the P3 and the error negativity (Ne). The target-locked N2 is a negative deflection with a fronto-central maximum, which has been linked to both the detection of stimulus novelty and cognitive control, in terms of response inhibition and the resolution of response conflict during responding (Folstein and Van Petten, 2008). It is thus thought to reflect the implementation of stimulus-response associations, i.e., response selection, which is hampered on interference or conflict trials (Gajewski et al., 2008). In the context of task switching paradigms, the N2 has been found to be decreased in latency and amplitude for task repetitions (Gajewski et al., 2010a). A training-related increase in N2 amplitude may thus reflect improved response selection. The subsequent target-P3, a positive deflection with a parietal focus, has been associated with the allocation of processing resources, specifically memory operations (e.g., Polich, 2007). As such, its amplitude is largest for single trials, intermediate for stay trials and lowest for switch trials (Kieffaber and Hetrick, 2005; Jost et al., 2008; Gajewski et al., 2010b; Gajewski and Falkenstein, 2012). A training-related increase in P3 amplitude can thus be interpreted in terms of improved resource allocation. Finally, the error negativity (Ne or error-related negativity, ERN) is an early negative deflection which is elicited by the detection of a response error (Falkenstein et al., 1991). A training-related increase in Ne amplitude thus reflects improvements in error monitoring.

In the present study, we wanted to corroborate and extend our previous findings on the functional neural processes mediating gains in executive functioning associated with multi-domain cognitive training in older adults. To this end, we employed a different task-switching transfer task than in our earlier study which featured two rather than three distinct tasks and introduced different levels of task difficulty. In order to gain more thorough insights into neural processes involved in task preparation and response selection we examined not only the ERP components described in our earlier study, but also the switch positivity during advance task preparation (Karayanidis et al., 2010) and the target-locked P2 (Kieffaber and Hetrick, 2005).

One-hundred and three healthy older adults were randomly assigned to either a multi-domain cognitive training group, an active control group receiving relaxation training or a passive no-contact control group. At pretest and posttest, we used a binary cue-based switch paradigm featuring two tasks with asymmetric difficulty levels as transfer task. Participants had to indicate either the font color or the word meaning of Stroop stimuli, that is color words which were printed in colored fonts which could either be congruent to the word meaning (i.e., the word “yellow” presented in yellow font) or incongruent to it (i.e., the word “red” presented in yellow font). Word reading is the dominant behavior in this context, rendering the color task much more difficult than the word task (Stroop, 1935). Previous research has indicated that age-related cognitive deficits can be exacerbated with increasing task difficulty (e.g., Bierre et al., 2016). As of yet, relatively little is known, however, about the impact of task difficulty on training and transfer gains in older adults. An fMRI study by Brehmer et al. (2011) has indicated that training-related benefits to neural efficiency may come to bear mainly under more difficult task conditions for this age group. The present study aimed to further examine this issue and pinpoint the specific neural processes which may benefit from multi-domain cognitive training under difficult as compared to easy task conditions. In order to do this, we examined two additional ERP components which were omitted in our earlier study on older adults (Gajewski and Falkenstein, 2012), the switch positivity and the target-locked P2. The cue-locked switch positivity has been linked to anticipatory processes associated with task-set reconfiguration. Its amplitude is thus highest for switch trials, intermediate for stay trials and lowest for single trials (Eppinger et al., 2007; Wylie et al., 2009; Jamadar et al., 2010; Karayanidis et al., 2010, 2011). The target-locked P2, an early positive deflection with a fronto-central focus, is reduced on switch compared to stay trials and has thus been related to the retrieval of stimulus-response bindings (Kieffaber and Hetrick, 2005; Schapkin et al., 2014).

Irrespective of task difficulty, we expected to observe performance gains from pretest to posttest in the multi-domain cognitive training group but not in the two control groups. In keeping with our earlier study (Gajewski and Falkenstein, 2012), we further expected that these performance gains would be accompanied by modulations of ERP components indexing the resolution of response conflict during response selection (N2) and the allocation of cognitive resources (target-locked P3) as well as the retrieval of stimulus-response bindings (P2) and anticipatory task-set reconfiguration (switch positivity). Based on previous research (Brehmer et al., 2011), we expected between-group differences to be more pronounced under difficult task conditions.

## Materials and Methods

### Participants

Participants were independently living, healthy older adults which were screened for sufficient visual and auditory acuity. Other exclusion criteria were a history of cardio-vascular, motor, oncological, psychiatric or neurological diseases. Participants were also excluded from the study if their self-reported cognitive training activity exceeded 1.5 h per week. As a result of this screening procedure, 32.5% of applicants were included in the study. After completing the pretest, the 114 participants were randomly assigned to a training group receiving a 4-month multi-domain cognitive training (remaining *N* = 32; 20 female, 65–82 years old, mean age: 70.5 years, seven drop-outs due to illness, relocation, technical malfunctions), a passive no-contact control group (remaining *N* = 37; 21 female, 65–88 years old, mean age: 70 years, two drop-outs due to technical malfunctions) and an active (social) control group receiving a 4-month relaxation training (remaining *N* = 34; 21 female, 65–87 years old, mean age: 70.9 years, two drop-outs due to illness). All groups were comparable with respect to age, education and cognitive status as assessed by Mini Mental State Examination (MMSE German version, Kessler et al., 2000), verbal IQ (MWT-B, Lehrl, 2005), forward and backward digit span and versions A and B of the Trail-Making Test (see, Wild-Wall et al., 2012, for details). All participants were included in the behavioral data analyses. Due to malfunctions of the EEG equipment, data from six participans (one of the cognitive control group, one of the active control group and three of the passive control group) could not be included in the ERP analyses. The study was carried out in accordance with the Declaration of Helsinki and with the recommendations of the local ethics committee of the Leibniz association. All participants gave written informed consent and received 100 Euro to recompense them for travel expenses.

### Multi-Domain Cognitive Training and Relaxation Training

Participants in the cognitive training and the active (social) control group completed two 90-min training sessions per week across a period of 4 months. Both trainings were conducted by payed professional trainers in small groups of no more than 12 participants. Participants who had missed regular sessions had the opportunity to take part in two additional sessions after the regular training had been completed. Participants were otherwise not encouraged to train outside of the regular training sessions.

Participants in the cognitive training group were first given basic information on cognitive functions, their relevance for activities of daily living and the impact of aging on these functions. Participants additionally learned memory strategies, such as the method of loci. Subsequently, participants completed 4 weeks of paper-pencil-based exercises focused on improving processing speed, selective attention, short-term memory span, verbal fluency and arithmetic and reasoning skills (sudokus; MAT, Lehrl et al., 1994; Klauer, 2008). Simultaneously, participants without prior computer experience were familiarized with the use of a computer mouse and keyboard. In the final cognitive training phase, participants completed computer-based cognitive exercises focused on perceptual speed, selective attention and memory (peds Braintrainer^1^; mentaga GYM^2^; Mental Aktiv^3^). This multi-domain cognitive training regimen did not include a task-switching task, a Stroop task or a combination thereof. A more thorough description of the multi-domain cognitive training has already been published elsewhere (see appendix of Gajewski and Falkenstein, 2012).

The relaxation training of the active (social) control group was comprised of gymnastic, back therapy, muscle relaxation and stretching exercises as well as techniques from autogenous training, progressive muscle relaxation, Qigong and massage therapy. The training also included elements of health education, giving basic information about healthy nutrition, the negative effects of addictive substances, such as alcohol and nicotine and the benefits of physical exercise.

### Pre- and Posttest Procedure

Before and after the interventions, participants completed pretest and posttest sessions, respectively. These included socio-demographic questionnaires (pretest only), paper-and-pencil-based neuropsychological tests and computer-based cognitive tests with concurrent EEG-recording. While the present study focuses on the Stroop switch task, data from other cognitive tasks has been reported elsewhere (Gajewski and Falkenstein, 2012; Wild-Wall et al., 2012). Cognitive testing and EEG-recording were conducted in a dimly lit, electrically-shielded and sound-attenuated room. All participants were tested individually and were seated 90 cm from a 15 inch CRT monitor with a resolution of 640 × 480. Stimulus presentation and response acquisition were controlled by an IBM-compatible computer running MS-DOS.

In the Stroop switch task, participants had to indicate either the font color or the word meaning of Stroop stimuli. Stroop stimuli (10 × 5–7 mm) were the German words “rot”, “grün”, “blau” and “gelb” (red, green, blue and yellow, respectively) which were presented on a black background in one of four colored fonts (red, green, blue and yellow). Fonts could either be congruent to the word meaning (i.e., the word “yellow” presented in yellow font) or incongruent to it (i.e., the word “red” presented in yellow font). Prior to the Stroop stimulus, participants were presented with a cue which indicated which task had to be performed in the current trial. A white square (37 × 37 mm) indicated that font color was the relevant stimulus dimension, whereas a white diamond (37 × 37 mm) indicated that participants had to respond to the word meaning. Responses were given by pressing one of four response keys which were mounted in a response box and each corresponded to a specific color.

A given trial thus began with the presentation of a fixation cross for 300 ms, followed by the presentation of the cue (diamond/square). After 1000 ms, the Stroop stimulus appeared within the cue and remained onscreen until the participant had responded by pressing one of the four response keys. Five-hundred milliseconds after the participant’s response a positive (plus sign) or negative (minus sign) feedback appeared. When the reaction time exceeded 2500 ms, the word “schneller” (faster) was presented in addition to the feedback in order to encourage participants to respond more quickly on subsequent trials.

Participants completed a total of 250 trials (50% congruent and 50% incongruent) in three distinct experimental blocks. The first two blocks were single blocks in which participants always had to perform either the word (52 trials) or the color task (52 trials). In the subsequent mixed block (146 trials), participants instead had to perform the color task (73 trials) or the word task (73 trials) in random order, as signaled by the cue. Thirty-six trials of each task type were stay trials in which the same task as in the preceding trial had to be performed. The other 37 trials were switch trials in which a different task than in the preceding trial had to be completed. Across all conditions, half of the trials featured congruent Stroop stimuli whereas the other half featured incongruent Stroop stimuli.

### Electrophysiological Recording and Analyses

EEG activity was recorded continuously from 32 active BioSemi Pin-Type electrodes arranged according to the extended 10–20 system in a preconfigured cap (Easy Cap, Easycap GmbH, Herrsching-Breitbrunn, Germany). Electrodes were placed at positions Fp1, Fpz, Fp2, F7, F3, Fz, F4, F8, FC3, FCz, FC4, T7, C3, Cz, C4, T8, CP3, CPz, CP4, P7, P3, Pz, P4, P8, PO3, POz, PO4, O1, Oz and O2. Eight additional electrodes were used to record the EOG and activity at the left and right mastoids. In the Bio-Semi system, ground and reference electrodes are replaced by a feedback loop between an active and a passive electrode at positions C1 and C2, respectively. Impedances for all electrodes were kept below 10 kΩ. Signals were digitized with a BioSemi Active Two amplifier at a sampling rate of 2048 Hz and a bandpass of 0.01–140 Hz.

For off-line analysis, data were downscaled to a sampling rate of 1000 Hz and digitally bandpass filtered at 0.05–17 Hz. The first trial of each experimental block and trials with an incorrect, very fast (<100 ms) or very slow (>2500 ms) response were excluded from further analyses. The EEG was segmented into cue-locked and target-locked epochs and baseline-corrected with respect to the 100 ms pre-stimulus interval. Vertical and horizontal ocular artifacts were corrected off-line (Gratton et al., 1983), while trials with other artifacts (maximum amplitude in the segment, ±150 μV; maximum voltage step between two successive sampling points, 50 μV; maximum difference between two sampling points within the segment, ±300 μV, lowest activity in a 100 ms interval, 0.5 μV) were excluded from averaging. Electrodes were re-referenced to linked mastoids. ERPs were averaged separately for each of the two tasks (color, word) and the three trial types (single, switch, stay).

The switch positivity and the target-locked P3 were quantified as mean amplitudes between 300 ms and 600 ms post-cue and post-stimulus, respectively, at electrode positions Fz, Cz and Pz. The target-locked P2 was quantified as the most positive local amplitude between 150 ms and 300 ms after stimulus onset at FCz. The subsequent target-locked N2 was measured as the most negative local amplitude between 200 ms and 400 ms post-stimulus at Cz. Electrode positions and time windows were selected on the basis of previous research (e.g., Polich, 2007; Folstein and Van Petten, 2008; Gajewski and Falkenstein, 2012; Schapkin et al., 2014).

## Results

### Behavioral Data

**Figure 1:**
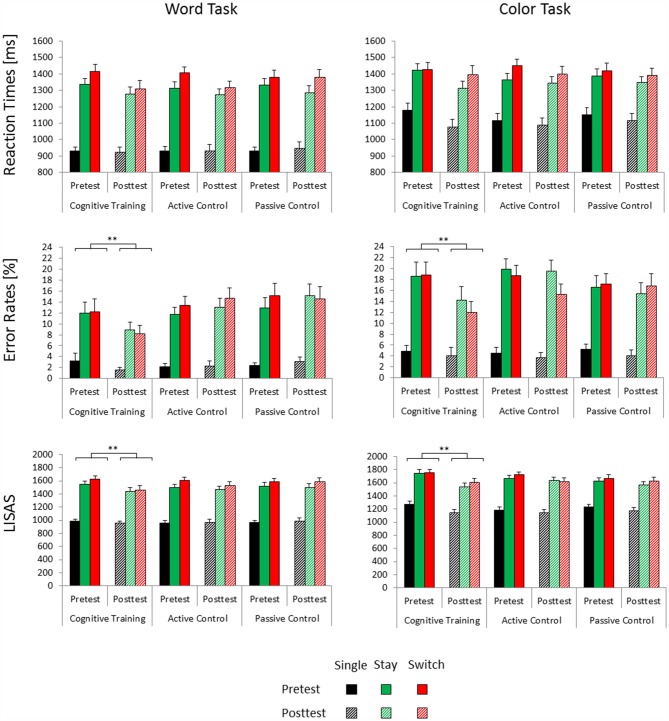
**Mean reaction times, error rates and linear integrated speed-accuracy score (LISAS) for the three groups (cognitive training, active control, passive control) as a function of session (pretest, posttest), trial type (single, switch, stay) and task (word task, color task).** Error bars indicate the standard error of the mean. Posttest reaction time gains associated with cognitive training are highlighted (**significant at *p* < 0.001).

The first trial of each experimental block was excluded from further analyses. Trials with an incorrect, very fast (<100 ms) or very slow (>2500 ms) response were not included in the reaction time analyses. Mean accuracy and reaction times as well as the linear integrated speed-accuracy score (LISAS = mean reaction times_condition_ + standard deviation of reaction times_total_/standard deviation of proportion of errors_total_ × proportion of errors_condition_; Vandierendonck, 2016) were computed (see Figure 1). Due to lack of errors, LISAS could not be computed for one participant from the cognitive training group. All behavioral parameters were analyzed in separate analysis of variances (ANOVAs) with the within-subject factors Test Time (pretest, posttest), Trial Type (single, switch, stay) and Task (color task, word task) and the between-subject factor Group (cognitive training, active control, passive control). For all parameters, we additionally computed mixing/general switch costs (stay trials − single trials) and specific/local switch costs (switch trials − stay trials) which were submitted to separate ANOVAs with the within-subject factors Test Time and Task and the between-subject factor Group. Results were Greenhouse-Geisser corrected, where appropriate. For the sake of brevity, we only list significant effects involving the factor Test Time. In order to specify training-related changes, these effects were further analyzed with Bonferroni *post hoc* tests.

In the reaction time data, we observed a significant main effect of Test Time (*F*_(1,100)_ = 8.21, *p* < 0.01, $\eta_{\text{p}}^{2}$ = 0.08) and a significant interaction of Test Time × Trial Type × Task (*F*_(2,200)_ = 5.48, *p* < 0.01, $\eta_{\text{p}}^{2}$ = 0.05). *Post hoc* tests indicated that for the color task, reaction times decreased from pretest to posttest on single and stay trials (both *p*s < 0.01), but not on switch trials (*p* = 0.18). In the word task, reaction time benefits were limited to switch trials (*p* < 0.01) and stay trials (*p* < 0.05), but did not emerge on single trials (*p* = 0.68). None of the effects involving interactions of the factors Test Time and Group were significant.

The accuracy data yielded a significant main effect of Test Time (*F*_(1,100)_ = 4.1, *p* < 0.05, $\eta_{\text{p}}^{2}$ = 0.04) and a significant interaction of Test Time × Group (*F*_(2,100)_ = 3.4, *p* < 0.05, $\eta_{\text{p}}^{2}$ = 0.06). Bonferroni *post hoc* tests showed reduced error rates at posttest compared to pretest only in the cognitive training group (*p* < 0.01), but not in the two control groups (both *p*s > 0.84). Baseline accuracy at pretest was equivalent for the three groups (all *p*s > 0.99).

For the LISAS data, we similarly found a significant main effect of Test Time (*F*_(1,99)_ = 12.21, *p* < 0.01, $\eta_{\text{p}}^{2}$ = 0.11) as well as a significant interaction of Test Time × Group (*F*_(2,99)_ = 3, *p* = 0.05, $\eta_{\text{p}}^{2}$ = 0.06). In keeping with the accuracy data, only the cognitive control group showed decreased LISAS, i.e., better performance, at posttest compared to pretest (*p* < 0.001, both control groups, *p*s > 0.18). At pretest, we observed no significant differences in baseline performance between the three groups (all *p*s > 0.89). The interaction of Test Time × Task (*F*_(1,99)_ = 6.01, *p* < 0.05, $\eta_{\text{p}}^{2}$ = 0.06) was also significant. *Post hoc* tests nevertheless showed significant performance benefits from pretest to posttest in both the color task (*p* < 0.001) and in the word task (*p* < 0.05).

Our analysis of mixing/general switch costs (stay trials − single trials) and specific/local switch costs (switch trials − stay trials) yielded no significant effects involving the factor Test Time for either accuracy, reaction times or LISAS.

### Behavioral Data Summary

Error rates and LISAS indicated performance gains from pretest to posttest only for the cognitive training group, but not for the two control groups. Training-related performance gains emerged for all trial types and could thus not be attributed to reductions of mixing/general switch costs or specific/local switch costs. All groups showed decreased reaction times at posttest compared to pretest. These unspecific practice effects were subject to the task participants had to perform: for the easier word task, reaction time benefits emerged under mixing conditions. For the more difficult color task, they were instead limited to single and stay trials which did not require a task set reconfiguration.

### ERP Data

The switch positivity and the target-locked P3 were analyzed in separate ANOVAs with the within-subject factors Electrode Position (Fz, Cz, Pz), Test Time (pretest, posttest), Trial Type (single, switch, stay) and Task (color task, word task) and the between-subject factor Group (cognitive training, active control, passive control). Target-locked P2 amplitude at FCz and N2 amplitude at Cz were analyzed in separate ANOVAs with the within-subject factors Test Time (pretest, posttest), Trial Type (switch, stay) and Task (color task, word task) and the between-subject factor Group (cognitive training, active control, passive control). Results were Greenhouse-Geisser corrected, where appropriate. Significant effects involving the crucial factor Test Time are listed and were further analyzed with Bonferroni *post hoc* tests.

### Cue-Locked Switch Positivity Amplitude

**Figure 2:**
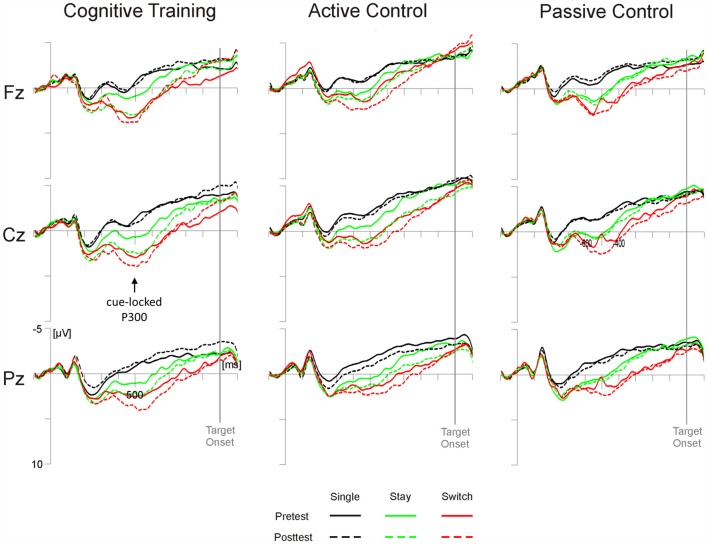
**Cue-locked grand average event-related potentials (ERPs) for the three groups (cognitive training, active control, passive control) at Fz, Cz and Pz as a function of session (pretest, posttest) and trial type (single, switch, stay).** Time scaling ranges from −100 ms to 1100 ms around cue onset and positive deflections are displayed downward. Cognitive training was associated with increased posttest amplitudes of the highlighted cue-locked P3 on stay trials (green lines).

Analyses of the switch positivity amplitude yielded a significant main effect of Test Time (*F*_(1,98)_ = 5.79, *p* < 0.05, $\eta_{\text{p}}^{2}$ = 0.06) as well as interactions of Test Time × Electrode Position × Trial Type × Task (*F*_(4,392)_ = 3.96, *p* < 0.01, $\eta_{\text{p}}^{2}$ = 0.04) and, crucially, Test Time × Group × Electrode Position × Trial Type (*F*_(8,392)_ = 4.83, *p* = 0.05, $\eta_{\text{p}}^{2}$ = 0.04). As illustrated in Figure 2, *post hoc* tests indicated that in the cognitive training group, stay trials elicited higher switch positivities at posttest compared to pretest at all sites (Fz and Cz, *p*s < 0.05; Pz, *p* = 0.05; all other *p*s > 0.14). In the active control group, higher posttest amplitudes emerged at Cz for stay trials (*p* < 0.05) and Pz showed a trend towards pre-post differences for single trials (*p* = 0.07; all other *p*s > 0.15). For the passive control group, on the other hand, we found no reliable pre-post differences at any electrode position (all *p*s > 0.29).

### Target-Locked P2 Amplitude and Latency

**Figure 3:**
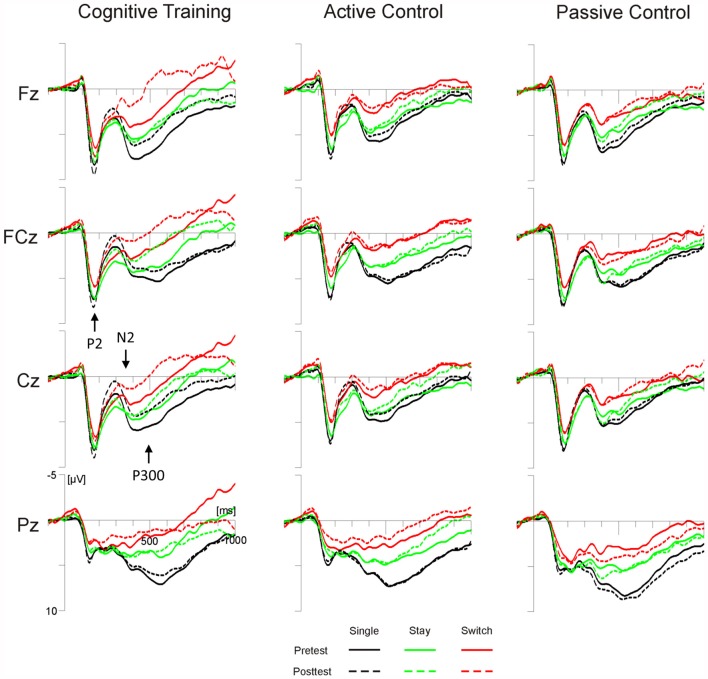
**Target-locked grand average ERPs for the three groups (cognitive training, active control, passive control) at Fz, FCz, Cz and Pz as a function of session (pretest, posttest) and trial type (single, switch, stay).** Time scaling ranges from −100 ms to 1000 ms around cue onset and positive deflections are displayed downward. Cognitive training was associated with increased posttest amplitudes of the highlighted N2 and decreased amplitudes of the highlighted P3 for all trial types. Single trials (black lines) additionally showed reduced latencies of the highlighted P2 at posttest.

For P2 amplitudes at FCz, we observed a significant interaction of Test Time × Task (*F*_(1,98)_ = 4.93, *p* < 0.05, $\eta_{\text{p}}^{2}$ = 0.05), yet *post hoc* tests indicated no significant changes from pretest to posttest for either the color or the word task (both *p*s > 0.11). None of the effects involving the factor Group reached significance. The P2 latency analysis yielded a significant main effect of Test Time (*F*_(2,196)_ = 4.6, *p* < 0.05, $\eta_{\text{p}}^{2}$ = 0.05) and a significant interaction of Test Time × Group × Trial Type (*F*_(4,196)_ = 2.42, *p* = 0.05, $\eta_{\text{p}}^{2}$ = 0.05). *Post hoc* tests showed reduced P2 latencies at posttest compared to pretest, but only on single trials of the cognitive training group (*p* < 0.01; all other *p*s > 0.14, see Figure 3).

### Target-Locked N2 Amplitude and Latency

The analysis of N2 amplitudes at Cz, yielded a significant interaction of Test Time × Group (*F*_(2,98)_ = 3.36, *p* < 0.05, $\eta_{\text{p}}^{2}$ = 0.06). *Post hoc* tests showed an increase in N2 amplitude from pretest to posttest for the cognitive training group (*p* < 0.01), but not for the two control groups (both *p*s > 0.34, see Figure 3). The N2 latency analysis showed a significant interaction of Test Time × Trial Type × Task (*F*_(2,196)_ = 6.25, *p* < 0.01, $\eta_{\text{p}}^{2}$ = 0.06). According to *post hoc* tests, N2 latencies were shortened from pretest to posttest only on stay trials of the color task (*p* < 0.01; all other *p*s > 0.13).

### Target-Locked P3 Amplitude

For the target-locked mean P3 amplitude, we observed significant interactions of Test Time × Electrode Position (*F*_(2,196)_ = 4.67, *p* < 0.05, $\eta_{\text{p}}^{2}$ = 0.05), and, importantly, Test Time × Group × Electrode Position × Trial Type (*F*_(8,392)_ = 2.72, *p* < 0.01, $\eta_{\text{p}}^{2}$ = 0.05). For both control groups, *post hoc* tests showed no significant amplitude differences between pre- and posttest, irrespective of trial type and electrode position (all *p*s > 0.21; see Figure 3). For the cognitive training group, on the other hand, P3 amplitudes decreased from pretest to posttest: at Fz, amplitude decreases were significant for single and switch trials (both *p*s < 0.01). At Cz, we observed significant decreases for switch and stay trials (both *p*s < 0.05) and a trend towards a decrease for single trials (*p* = 0.07; Pz, all *p*s > 0.39).

### ERP Data Summary

For all trial types, cognitive training was associated with an increase of the target-locked N2 and a subsequent amplitude decrease at fronto-central locations in the time range of the target-locked P3. On single trials, we additionally observed a training-related reduction of target-locked P2 latencies. In the cue-target interval, the switch positivity on stay trials was increased at posttest for both the cognitive training group and the active control group. The passive control showed no significant ERP differences between pretest and posttest.

## Discussion

The present study was aimed at examining the effects of multi-domain cognitive training and task difficulty on executive functions in older adults. To this end, healthy older adults were randomly assigned to a passive control group, an active control group receiving 4 months of relaxation training and a cognitive training group who completed 4 months of multi-domain cognitive training. In pre- and posttest sessions, we recorded behavioral and ERP indices of performance in an untrained task switching paradigm featuring Stroop stimuli. Participants had to attend two tasks with different difficulty levels, an easier word reading task and a more difficult color naming task (see Stroop, 1935).

At the behavioral level, we observed reduced reaction times at posttest compared to pretest. Although this effect appeared to be driven mainly by reaction time reductions in the cognitive training group and the active control group, it was not significantly modulated by the factor Group. It thus should be considered a practice effect induced by repeated testing rather than a performance gain related to the increased activity associated with training regimen. Interestingly, this practice effect was modulated by task difficulty: in the easier word-task, reaction time benefits emerged on stay and switch trials, but not in single task blocks. In the more difficult color task, reaction times instead decreased for single and stay trials, but not for switch trials. In light of the low difficulty level of the word task, it is feasible that only the more complex mixed block offered room for improvement because performance for the less demanding single trials was already at ceiling at pretest. For the more difficult color task, on the other hand, the task set configuration associated with switch trials may have further elevated the difficulty level to such a degree that practice was not sufficient to generate reliable reaction time benefits.

In the context of the present study, accuracy and LISAS results were more noteworthy than the reaction time data as they indicated performance benefits associated exclusively with multi-domain cognitive training: both parameters were reduced at posttest compared to pretest only for the cognitive training group, but not for the two control groups. This result pattern corroborates and extends previous research on multi-domain cognitive training which could show beneficial transfer effects to executive functions in younger, middle-aged and older adults (e.g., Gajewski and Falkenstein, 2012; Baniqued et al., 2015; Gajewski et al., 2017). In keeping with these studies, training-related performance gains became manifest in increased response accuracy. In contrast to our initial hypothesis, however, these accuracy improvements were equivalent in the color and in the word task.

Cognitive training was not only associated with gains in response accuracy, but also with modulations of cue- and target-locked ERPs from pretest to posttest. Whereas the switch positivity in the task preparation period was larger at posttest both in the cognitive training group and the active control group, changes in target-N2 and target-P3 amplitude as well as target-P2 latency were limited to the cognitive training group. Like the training-related benefits in accuracy, these ERP modulations were not subject to the task participants had to perform.

ERPs in the cue-target interval showed modulations from pretest to posttest after either type of training regimen: on posttest stay trials, we observed an increase in switch positivity which was widespread for the cognitive training group and limited to central sites for the active control group, but absent in the passive control group. Modulations of the switch positivity are thought to reflect the degree of task set updating necessary to prepare for the upcoming task (for a review see Karayanidis et al., 2010). An enhancement of switch positivity amplitude from pretest to posttest, notably in stay trials, may thus indicate a training-related boost to the efficiency of maintaining a task set from one trial to the next under mixing conditions. As both the cognitive training group and the active control group showed an increase in switch positivity amplitude, this efficiency gain may be due to unspecific vitalization associated with training regimen in general (see Gajewski and Falkenstein, 2015a).

Regarding the target-locked ERPs, the present target-P2 data indicate that multi-domain cognitive training has the potential to accelerate the processing operations underlying the P2, at least under single task conditions. Previous research has linked the P2 to the retrieval of stimulus-response bindings (Kieffaber and Hetrick, 2005; Gajewski et al., 2008; Schapkin et al., 2014). The subsequent target-locked N2 has been associated with cognitive control processes, such as response inhibition, the resolution of response conflict and response selection (for a review see Folstein and Van Petten, 2008). The training-related enhancement of the target-locked N2 we observed in the present study is consistent with our previous reports (Gajewski and Falkenstein, 2012; Gajewski et al., 2017): multi-domain cognitive training, was previously associated with an increase in target-locked N2 amplitude in cue-based and memory-based versions of a task switching paradigm featuring three tasks with comparable difficulty levels. Whereas the earlier study showed reliable N2 enhancements mainly for switch trials, the present N2 data as well as data from the later study indicate that cognitive training can also lead to amplitude increases on single and stay trials (see Gajewski and Falkenstein, 2015a,b, for similar N2 enhancements due to habitual physical activity).

Taken together with our previous findings (Gajewski and Falkenstein, 2012; Gajewski et al., 2017), the present ERP data thus corroborate the idea that multi-domain cognitive training can benefit processes involved in response selection, especially in older adults. At the behavioral level, such a training-related improvement of response selection consistently appears to translate into an improvement of response accuracy not only in older adults (the present study, Gajewski and Falkenstein, 2012) but also in younger participants (Gajewski et al., 2017). To be more specific, the present and previous data suggest that target-P2 and target-N2 are related to the retrieval or activation of stimulus-response mappings or task sets (target-P2) and the implementation of these sets (target-N2). The mechanisms reflected in these ERP components are thus essential for successfully executing a task-appropriate reaction, i.e., for pressing the correct response key. When this process is enhanced as indicated, for example, by a negative shift in the target-locked N2, participants are less likely to make an error. In other words, cognitive training enhances the ability to make a correct decision. For the present study, this was the case irrespective of task difficulty and on both switch and non-switch trials.

In contrast to our earlier report on older adults (Gajewski and Falkenstein, 2012), the present data show an amplitude decrease in the time window of the target-locked P3 at fronto-central electrodes following cognitive training. In the earlier study, participants had instead shown a training-related increase in target-locked P3 amplitudes at posterior sites which we interpreted in terms of improved cognitive resource allocation. In younger adults, the P3 usually has a clear-cut parietal focus (for a review see Polich, 2007). For the present older participants, we instead observed cue-locked and target-locked P3s with a more widespread distribution featuring parietal and frontal foci. This is in line with previous age-comparative research which has indicated that older adults may show a broader distribution of the P3 which extends to anterior sites as well and which likely reflects the compensatory increased recruitment of prefrontal brain areas involved in cognitive control (Kray et al., 2005; Eppinger et al., 2007; Adrover-Roig and Barceló, 2010; Kopp et al., 2014). As the target-P3 amplitude decrease observed in the present study was limited to fronto-central electrodes, it could potentially reflect a training-related reduction in this compensatory over-recruitment of frontal areas. Alternatively, the target-P3 amplitude decrease could be related to the enhancement of the preceding fronto-central target-N2 which extends into the P3 peak latency range, or to an even broader negative shift in the time range of both target-locked N2 and P3 (see Gajewski and Falkenstein, 2015a,b). Further ERP research on cognitive training in older adults is needed to clarify this issue.

Previous research on the impact of transfer task difficulty on training-related performance gains is scarce. In one of the few studies on the subject, Brehmer et al. (2011) examined the performance of a cognitive training group and an active control group in a working memory transfer task featuring high and low load conditions. They found that neither group showed performance gains from pretest to posttest in either condition suggesting that performance was already at ceiling at pretest. The present study instead featured a more difficult transfer task which offered room for improvement from pretest to posttest. Under these conditions, practice effects which were not directly associated with cognitive training were subject to task difficulty whereas genuine training-related performance benefits were not. Likewise, cognitive training benefited performance on single, stay and switch trials to a similar degree. As a result, we were unable to link training-related performance gains to reductions of mixing/general switch costs or specific/local switch costs as previous studies have done (e.g., Gajewski and Falkenstein, 2012). Note, however, that particularly specific/local switch costs were minimal at baseline in the cognitive training group. Any potential impact of multi-domain cognitive training on these costs thus would have been difficult to detect, in the present study.

### Conclusions

The present study corroborates and extends our understanding of the neural underpinnings of performance gains associated with multi-domain cognitive training in older adults. A 4 month multi-domain cognitive training had beneficial effects on response accuracy in an untrained binary switch paradigm featuring two tasks with distinct difficulty levels. These training-related performance gains were likely mediated by an increase in target-locked N2 amplitude, an amplitude reduction in the time range of the target-locked P3 and a decrease in target-P2 latency. These ERP modulations indicate benefits to neural processes involved in response selection which resulted in reduced error rates on both switch and non-switch trials. Our findings suggest that multi-domain cognitive training increases slow negative potentials during target processing which enhance the N2 and may additionally reduce the amplitude of a subsequent P3-like component on both switch and non-switch trials and irrespective of task difficulty.

## Author Contributions

KK conducted the data analysis and wrote the manuscript. PDG and MF were involved in study conception and data acquisition and revised and approved the manuscript. CF was involved in study conception as well as the acquisition and analysis of the data and revised and approved the manuscript.

## Conflict of Interest Statement

The authors declare that the research was conducted in the absence of any commercial or financial relationships that could be construed as a potential conflict of interest.

## Footnotes

^1^www.ahano.de

^2^www.mentage.com

^3^www.mental-aktiv.de
